# Attention switching through text dissimilarity: a cognition research on fragmented reading behavior

**DOI:** 10.3389/fnhum.2024.1402746

**Published:** 2024-06-25

**Authors:** Jingjing Cao, Jingtao Luo, Jia Zhou, Yunshan Jiang

**Affiliations:** ^1^School of Management Science and Real Estate, Chongqing University, Chongqing, China; ^2^College of Mechanical and Vehicle Engineering, Chongqing University, Chongqing, China

**Keywords:** fragmented reading, attention switching, text dissimilarity, working memory load, working memory capacity, P200

## Abstract

People tend to obtain information through fragmented reading. However, this behavior itself might lead to distraction and affect cognitive ability. To address it, it is necessary to understand how fragmented reading behavior influences readers’ attention switching. In this study, the researchers first collected online news that had 6 theme words and 60 sentences to compose the experimental material, then defined the degree of text dissimilarity, used to measure the degree of attention switching based on the differences in text content, and conducted an EEG experiment based on P200. The results showed that even after reading the fragmented text content with the same overall content, people in subsequent cognitive tasks had more working memory capacity, lower working memory load, and less negative impact on cognitive ability with the text content with lower text dissimilarity. Additionally, attention switching caused by differences in concept or working memory representation of text content might be the key factor affecting cognitive ability in fragmented reading behavior. The findings disclosed the relation between cognitive ability and fragmented reading and attention switching, opening a new perspective on the method of text dissimilarity. This study provides some references on how to reduce the negative impact of fragmented reading on cognitive ability on new media platforms.

## 1 Introduction

With digitization, reading, a common interaction between people and media, has profoundly changed. The so-called new media relies on digital means to communicate (Szabo, 2014). Its platform refers to the online media platform where users could browse mass information shared by other users (Zhuang et al., 2023). The characteristic of fragmented reading, which is carried out by readers through various new media, is rapid attentional shifts from one short content to another (Zhang, 2012). Specifically, content fragmentation included two main characteristics of low continuity and high dispersion, which referred to the shorter and more dissimilar content of texts (Xie, 2019). These new media platforms have further strengthened the characteristics of content fragmentation, and the impact of fragmented reading on users is becoming more and more common.

Although fragmented reading could enable users to quickly and efficiently read the content, users’ attention quickly switches between these fragmented contents without in-depth thinking (Liu and Huang, 2016), and this reading mode might cause inertia and atomized thinking (Xie, 2021). Meanwhile, the attention switching between different tasks, and attention needs to be continuously switched and refocused (Uncapher et al., 2017; Madore et al., 2020), which might cause distraction and greater susceptibility to interference from irrelevant stimuli in the external environment (Ophir et al., 2009). These effects might be similar to the effects of media multitasking on cognitive ability, which might be caused by frequent attentional switching or shifting (Madore et al., 2020).

Some researchers have explored the cognitive impact of users’ attention switching between multiple tasks (Ophir et al., 2009), which might be similar to the “attention switching” of fragmented reading behavior in new media platforms. With frequent shifts between short content and another, cognitive conflicts will occur frequently (Egner et al., 2007), and weaken the brain’s ability to process cognitive signals and executive function. If this state continues, it will inevitably disrupt attention control (Clapp et al., 2011), working memory and other mechanisms (Diamond, 2013), and cognitive flexibility also will be weakened, resulting in the solidification of thinking and the reduction of creativity (Blackwell, 2013). However, in contrast to media multitasking, attention switching, which occurs in the brain during fragmented reading, is difficult to show through external behavior. More importantly, unlike media multitasking, where there are easily distinguishable physical boundaries, the differences in text content are more thematic or semantic in fragmented reading. Therefore, it is difficult to conduct quantitative research and intervention on fragmented reading behaviors in the new media platforms, and relevant studies are often used by questionnaire surveys and qualitative analysis.

To understand what is at play, this study analyzed the differences between fragmented reading behaviors and media multitasking in new media platforms. Then, this study determined a measure of attention switching in fragmented reading behavior, which mentioned the new method to the text dissimilarity based on the characteristics of content fragmentation. Further, an electroencephalogram (EEG) experiment was conducted to explore the relationship among the degree of text dissimilarity, attention switching, and working memory, and explore how attention switching affects people’s cognitive ability. The results would help researchers better understand the cognitive ability mechanism of fragmented reading behavior.

## 2 Literature review

### 2.1 The attention system of the human brain

Attention is a cognitive process of selectively focusing on all perceptible stimuli or information while filtering out irrelevant or distracting factors. The characteristics of attention are active nature and selectivity. Attention reflects the concentration of awareness of some phenomenon to a certain stimulus or target. Concentration (Astle and Scerif, 2008), in psychology, reflects the process in which human cognitive activities continuously pay attention to the selective object or make discrete responses to specific stimuli (Neo and Chua, 2006; Reed, 2012). The former is called sustained attention (Esterman and Rothlein, 2019), also named vigilance (Warm et al., 2008), while the latter is called focused attention. The process of attention depends on the level of human arousal and also needs the construction of relevant neural circuits (Petersen and Posner, 2012). Appropriate arousal levels are critical to attention, and a growing body of evidence, which contains modern physiology and pharmacology, suggests that the locus coeruleus is most critical in the regulation of arousal levels (Petersen and Posner, 2012). Some studies show that low tonic LC activity is associated with low task engagement due to low arousal, while high tonic LC activity results in low task engagement due to high arousal and distractibility. Both two levels of arousal are associated with weak responses to task-related stimuli, which could be described by a classic inverted U-shaped relationship between task performance and arousal level (Aston-Jones and Cohen, 2005; Unsworth and Robison, 2017). Although arousal forms the physiological basis of attention formation, for a given cognitive task, the level of performance or performance of the task is also related to how attention resources are used. In terms of the construction of relevant neural circuits, current research focuses on how neural networks composed of different brain regions affect each other, a process that is part of the executive control function in cognitive psychology (Ralph et al., 2014).

Attention is the cognitive process of selectively focusing on specific stimuli or information while filtering out irrelevant or distracting factors. Research related to the attention also includes studies on mind wandering. Broadly speaking, mind wandering refer to during a task refers to stimuli or thoughts unrelated to the task that impair the performance of the ongoing task (Christoff et al., 2016; Seli et al., 2018). Mind wandering is generally classified as intentional and unintentional, both of which might be influenced by the difficulty of the task. Intentional mind wandering tended to decrease with increasing task difficulty, while unintentional mind wandering was the opposite pattern (Seli et al., 2018). The viewpoints on mind-wandering in research primarily involve three models: *the Resource Control Theory* (Thomson et al., 2015), *the Opportunity Cost Model* (Kurzban et al., 2013), and *the Information Processing Theory* (Etzel et al., 2015; Qiao et al., 2017). The first two models both considered attention as a limited resource. The Resource Control Theory suggests that the brain areas, related to attention control and mind-wandering, fluctuate over time based on task performance, and these fluctuations should be negatively correlated (Jackson et al., 2017). The opportunity cost model explains the mind-wandering from the perspectives of motivation, value, and reward (Kurzban et al., 2013). Additionally, task performance-based rewards could enhance attentional focus (Esterman et al., 2016; Massar et al., 2016). The third model suggested that attention could facilitate the representation of task-related stimuli and this representational communication by brain neural networks (Esterman et al., 2014; Ki et al., 2016), and the optimal state of attention might be low-energy and relaxed, which is related to the concept of “flow” (Fortenbaugh et al., 2018).

Considering simultaneously focusing on multiple cognitive tasks, is known as divided attention. Numerous studies have indicated that divided attention could reduce memory effects (Murdock, 1965; Barnes and Dougherty, 2007), and individuals might underestimate the extent of memory decline (Finley et al., 2014). The competition theory suggests that attention comes from the victory of stimuli in different levels of sensory systems, and intentional focus on the target (endogenous orienting) seemed to reduce the impact of competing stimuli (Desimone, 1995). Compared to automatic exogenous orienting, endogenous orienting appeared to activate a more extensive cortical network to achieve the internal transfer of attention resources (Mighter et al., 2004).

### 2.2 Sequential multitasking: attention switching

Research on attention under multitasking could be roughly divided into two types: one is to study how attention is allocated during multitasking (Senders, 1964; Wickens et al., 2017), and the other is to study the impact of multitasking on attention in cognitive ability (Alzahabi and Becker, 2013; Yeykelis et al., 2014). The former focused on the factors affecting attention allocation in the operating system and the relationship between attention and job performance (Wickens and Alexander, 2009), while the latter focuses on the switching cost of attention in media multitasking and the impact of long-term media multitasking on cognitive abilities such as attention and working memory (Nikkelen et al., 2014; Ralph et al., 2015).

Research on human–machine system attention allocation originated in the field of aviation, which primarily focused on how pilots allocated attention across multiple dashboards or display areas (Carrier et al., 2009). Building on studies of attention allocation by flight, Wickens first introduced the model to investigate how drivers allocated visual attention to “Areas of Interest” (AOI) on different screen display areas (Senders, 1964). He proposed four primary factors influencing attention allocation: Salience, Effort, Expectancy, and Value, collectively known as the SEEV model. The value factor was subjectively assessed based on different tasks (Wickens and Alexander, 2009).

Noticing SEEV (N-SEEV) expands upon the SEEV model, distinguishing between static visual attention and dynamic visual attention (Steelman-Allen et al., 2009). Subsequent research constructed a model for attention allocation under multiple factors and provided quantifiable evidence. Unlike the sequential tasks in the SEEV models, the Strategic Task Overload Management (STOM) model studies attention-switching behaviors while concurrently performing two or more tasks (Wickens et al., 2016).

Some multitasking researchers have found that individuals’ cognitive resources might be limited, and simultaneous execution of multiple tasks is not feasible. Therefore, media multitasking requires attention to continuously switch between various media resources, and there is a switching cost when attention shifts between different tasks (Uncapher et al., 2017; Madore et al., 2020).

Regarding attention dispersion, this might be because heavy media multitaskers employed more attention to multiple media, and were unable to exclude the influence of irrelevant stimuli in the environment (Ophir et al., 2009). Cain and Mitroff pointed out that in tasks with high attention demands, heavy media multitaskers had more scattered attention and processed more irrelevant stimuli than light media multitaskers (Cain and Mitroff, 2011). It seems that heavy media multitaskers have a broader attentional scope and are more susceptible to environmental interference (Ralph et al., 2015). Yap and Lim (2013) demonstrated that heavy media multitaskers tended to maintain multiple attentional focuses, while light media multitaskers tended to maintain a single attentional focus (Ralph et al., 2015). In tasks requiring sustained attention, it is challenging for heavy media multitaskers to focus their attention (Rosen et al., 2013). Additionally, in terms of memory performance, heavy media multitaskers were associated with poorer working memory performance (Uncapher et al., 2017), and possibly because of more interference affecting the encoding of target information, they also seemed more forgetful (Madore et al., 2020). However, heavy media multitaskers had lower switching costs (Alzahabi and Becker, 2013).

Regarding attention switching, media multitasking requires individuals to continuously switch attention between different tasks. In this frequent switching process, individuals’ arousal levels might increase, and heavy media multitaskers might be more accustomed to high-arousal environments (Yeykelis et al., 2014). When faced with low arousal activities, they might need a large amount of immediate stimuli to maintain the previous high arousal state. Therefore, attention issues might arise in low-arousal situations (Nikkelen et al., 2014).

In addition, studies by Lillard and Peterson (2011) found that fast-paced stimuli could bottom-up trigger individuals’ attention orientation. Ralph et al. (2014) pointed out that excessive cognitive behavior triggered by bottom-up external stimuli might damage executive control functions. Therefore, fragmented reading behavior could hurt cognitive abilities, including attention. This impact is likely to occur in the higher stages of cognitive processes, namely, the conceptual cognition of cognitive content or the maintenance process of working memory representations.

## 3 Experimental hypotheses

Fragmented reading behavior and media multitasking on new media platforms have similar effects on cognitive ability, potentially both caused by attention switching (Madore et al., 2020). However, significant differences exist in the underlying cognitive mechanisms between the two behaviors. Media multitasking refers to the act of simultaneously engaging in different tasks across multiple media platforms (Ophir et al., 2009) and the process has multiple focuses of attention. In contrast, fragmented reading behavior might have one focus of attention (Feng et al., 2019), where the frequent orientation of attention and distraction of attention might not occur, yet the negative impacts on attention persist (Blackwell et al., 2009). Therefore, this impact is most likely to occur at the higher stages of cognitive processes, specifically during the process of cognitive concepts or working memory representations.

The construction of cognitive processes stems from the activation and inhibition of brain regions. As external stimuli are processed by the brain, attention allocates cognitive resources to focus on aspects of information (Fisher et al., 2018), requiring retrieval of relevant knowledge from short-term or long-term memory and activation of corresponding brain regions (Rauss and Pourtois, 2013). The shift of cognitive tasks in fragmented reading behavior appears to result from the inhibition of neurons previously activated for the preceding cognitive content, while neurons necessary for the current cognitive content are activated. Attention swiftly shifts between different textual content, potentially indicating the continual maintenance of new information chunks in working memory.

We hypothesize that the degree of content dissimilarity in fragmented reading behavior may be related to the extent of attention switching, which might be caused by the differences in conceptual cognition or working memory representation. This means that the degree of text dissimilarity in cognitive content before and after might be related to the degree of consumption of attention resources or cognitive resources, and might also affect cognitive ability in subsequent tasks (work or study). In addition, a comparative investigation of assessment of brain activities in the information different degree during the proceeding and subsequent content might also be limited by the length of working memory.

Therefore, the research will propose the following hypotheses based on the text dissimilarity degree between the two kinds of texts:

H1: Compared with reading the text content with a lower degree of text dissimilarity, when people perform subsequent cognitive tasks, reading higher texts will have less working memory capacity.

H2: When people read the text content with a higher degree of text dissimilarity, the readers in subsequent cognitive tasks had more working memory load than lower degrees.

H3: In the reading process, reading the text content with a higher degree of text dissimilarity will consume more attention resources than a lower degree.

A cognitive experiment was designed in this study to test the above hypotheses.

## 4 Experimental design

### 4.1 Independent variable

In this experiment, a group of reading materials with different levels of text dissimilarity was used as an independent variable. As mentioned above, this research mainly examines the influence of the degree of text dissimilarity between Chinese texts on working memory and attention during reading text. First of all, the textual dissimilarity in the process of text reading is dynamic, so this suggests that people need to compare the current reading materials with the read text materials that they have read in their memory. However, individuals have a limited working memory capacity to remember only a limited number of blocks of information, which means that comparing current reading with all previous reading material does not yield meaningful results. Therefore, this paper defines the degree of text dissimilarity as follows:

For a text being read, the text dissimilarity (diff*_i_*) calculation method is as follows:


(1) $${\mathrm{diff}}_{i}=\overset{i-1}{\underset{k=i-\mathrm{span}+1,0}{\sum }}f\left(D_{k},D_{i}\right)$$

*D_i_* indicates the text that is currently being read, and *D_k_* indicates the text that was previously read. *f*(*D_i_*, *D_k_*) represents the function of the text dissimilarity. In the experiment, the subject terms between two texts are consistent, the result is 0, and the subject words are inconsistent, the result is 1. span represents the length of working memory and is used to limit the range of text comparisons and is set to 7 in Eq. 1.

For a group of texts, the degree of text dissimilarity (Diff) was calculated by Eq. 2, in which *i* represents that the text has currently been read and *N* is the number of read texts:


(2) $$\mathrm{Diff}=\overset{N}{\underset{i=1}{\sum }}{\mathrm{diff}}_{i}$$

This experiment needs to consider the requirements of experimental design, experimental time, and the endurance of subjects, so this research design that a group of reading contents is composed of 60 sentences, which includes six topics (span − 1), and 10 pieces of text are selected in each topic.

To explore the text dissimilarity of 60 sentences, the distribution of Diff was investigated in this experiment, which is calculated using the Eq. 2. Each sentence is marked with a particular subject type, the order of each sampling is randomly scrambled, and the different degree of text dissimilarity is calculated once. A total of 1,000,000 samples are taken in a group of reading texts.

To clearly distinguish the varying degrees of text dissimilarity in the experiment, the three-sigma was used to select the variable level, and the range of 275 to 300 covers most cases. Therefore, we refined that a degree, less than 275, was regarded as the low difference degree and recorded as “min”; and the difference degree of text dissimilarity greater than 300 is taken as the level of high text dissimilarity and marked as “max.” Additionally, the degree of text dissimilarity of a long article (assuming only one topic, artificially divided into 60 texts) is set to 0, and the degree of text dissimilarity is 0 as the baseline level, recorded as the baseline. Taking the degree of text dissimilarity as an independent variable, there are three levels in total, as shown in Table 1.

**Table 1:** Levels of text dissimilarity in a group of reading materials.

Levels of text dissimilarity	The number of subjects	The number of texts	Condition
max	6	60	>300
min	6	60	<275
baseline	1	60	0

### 4.2 Dependent variables

As mentioned above, attention switching might affect cognitive ability in subsequent tasks, especially working memory and executive control functions, which are closely related to the completion of cognitive tasks (Blackwell et al., 2009; Diamond, 2013). In addition, attention to task-switching between multiple media in media multitasking behavior has caused switching costs and loss of additional attention resources (Ophir et al., 2009). Whether attention switching has the same pattern and measuring the cost of switching is also the focus of this paper. Therefore, based on the three hypotheses, the experiment selected three dependent variables: working memory capacity, working memory load, and attention resources. They are described as follows:

#### 4.2.1 Working memory capacity

The digital span task was used to measure the participants’ working memory capacity. The size of the working memory capacity reflects the efficiency of the executive function, and this task is commonly used for such measurements.

The task of the digital span test is usually 10 digits (0 to 9). This is because the test results using numbers are not affected by semantics change, frequency of occurrence in everyday life, complexity, and other factors, and could better evaluate a participant’s working memory capacity (Jones and Macken, 2015).

**Figure 1:**
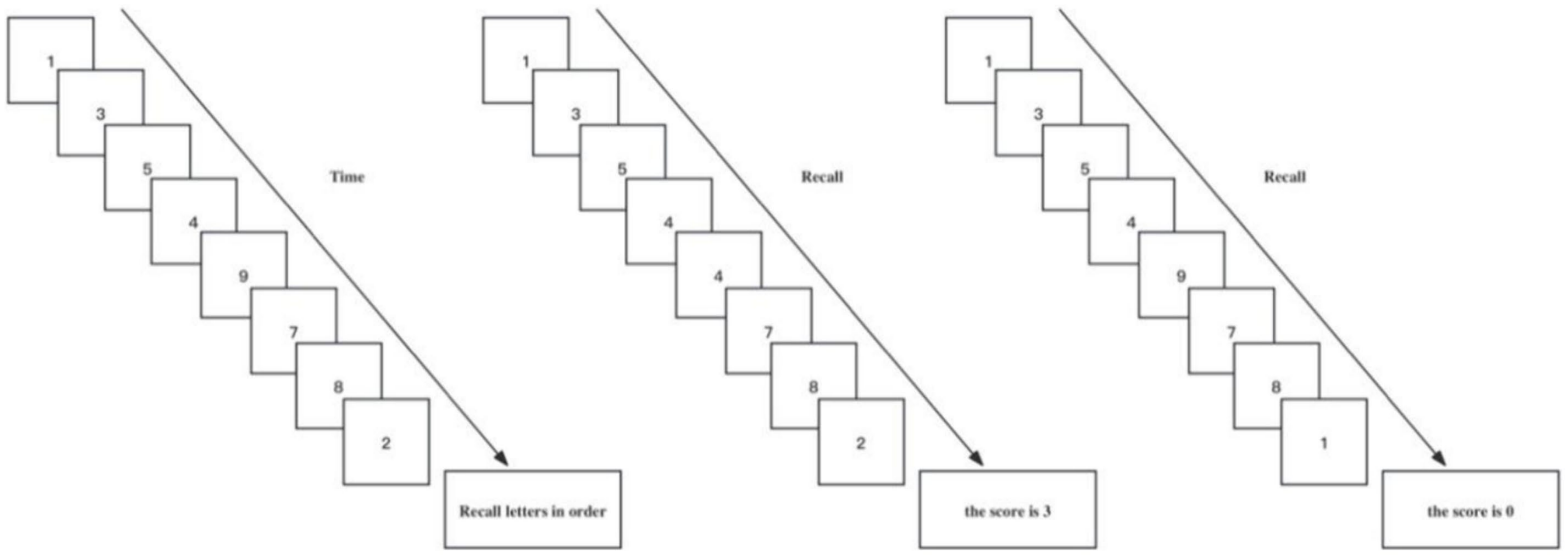
The digit span task testing process.

As shown in Figure 1, in the digital memory span test task, participants are presented with a series of numbers, and one number occurs only once time. The sequence of numbers is longer and longer, and two consecutive numbers are not the same. After the number was displayed, the participants were asked to recall the numbers in reverse order of their appearance, and the correct number length consecutively recalled as a result of the number memory span task. If the last number that appears is not correctly recalled, the result of the test is 0.

#### 4.2.2 Working memory load

The working memory load was examined by the power spectral density (PSD) in theta band EEG (Electroencephalogram). Working memory load refers to the amount of working memory resources used. When the working memory load is too high, it is difficult for people to act in response to new external stimuli or effectively extract the long-term memory storage in the brain, which is related to the current task, and this results in a decline during the task performance and an increased possibility of error in the task performance.

Working memory seems to be closely involved with the prefrontal cortex, and working memory load is closely related to the theta oscillations in the prefrontal cortex. Memory tasks could induce significant theta oscillations, and with the increase of working memory load, the theta oscillatory activities also increase (Zhang, 2012).

#### 4.2.3 Attentional resources

In the experiment, the average amplitude of the ERP (event-related potential) P200 component was used to measure attention resources. The P200 component, which might be part of the cognitive matching system, is distributed around the brain of the centro-frontal and the parieto-occipital areas and is usually found to be most pronounced in the frontal scalp region. The P200 component might be related to the process by which the brain uses contextual information to analyze upcoming stimuli and compare them with information stored in memory (Federmeier, 2002; Wlotko and Federmeier, 2007). In the Odd Ball paradigm, unpredictable auditory stimuli could induce significant positive deflection components in the event-related potentials (Squires et al., 1975). The latency of this component reflects the reaction time needed by the subject to distinguish between the exotic and non-exotic stimuli (Picton, 1992). The average amplitude of this component reflects the amount of attentional resources or the degree of attentional focus exhibited by the participants. The higher the average amplitude of the positive deflection component induced by the simple singular stimulus, the more attention resources or attention concentration (Ferrari et al., 2010).

## 5 Experimental materials

Based on the independent variable design outlined in Section 4.1, this study selected a set of six distinct themes for the experiment. To accentuate the divergence between these themes, this paper determines six topics, which refer to the news classification of Toutiao. Under different topics for news, the corresponding news text is selected according to the theme words, and the text content must include the designated theme words. Each theme selects 2 theme words, a total of 12 theme words, with details provided in Appendix A.

Because a quantity of EEG trials should be included in an experiment to obtain a stable and reliable version of a given ERP component, this experiment should comprehensively consider the number of trial requirements, experiment time, and participants’ endurance. Each participant in one experiment need read four groups of experimental materials at intervals, in which a group contained 60 texts and each theme word was associated with 10 texts. Refer to Appendix B for a detailed overview of the selected materials for this experiment.

The six topics were randomly sorted and labeled, and the subject words were randomly sorted and assigned to the four groups of experimental texts. To mitigate the potential influence of these words, the theme words were maintained the same between the first and third experimental text sets, as well as in the second and fourth sets. The outcomes of the randomized arrangement of themes and theme words can be shown in Appendix C.

According to the degree of text dissimilarity designed in Section 4.1, the text is arranged by requirements. To control the impact of text order, a topic word is randomly selected and ensures its consistent order of appearance in both two conditions, which is used as the “oddball” stimulus in the oddball paradigm. The resulting text arrangement results are marked by subject plus number. See Appendix C for text arrangement results.

**Figure 2:**
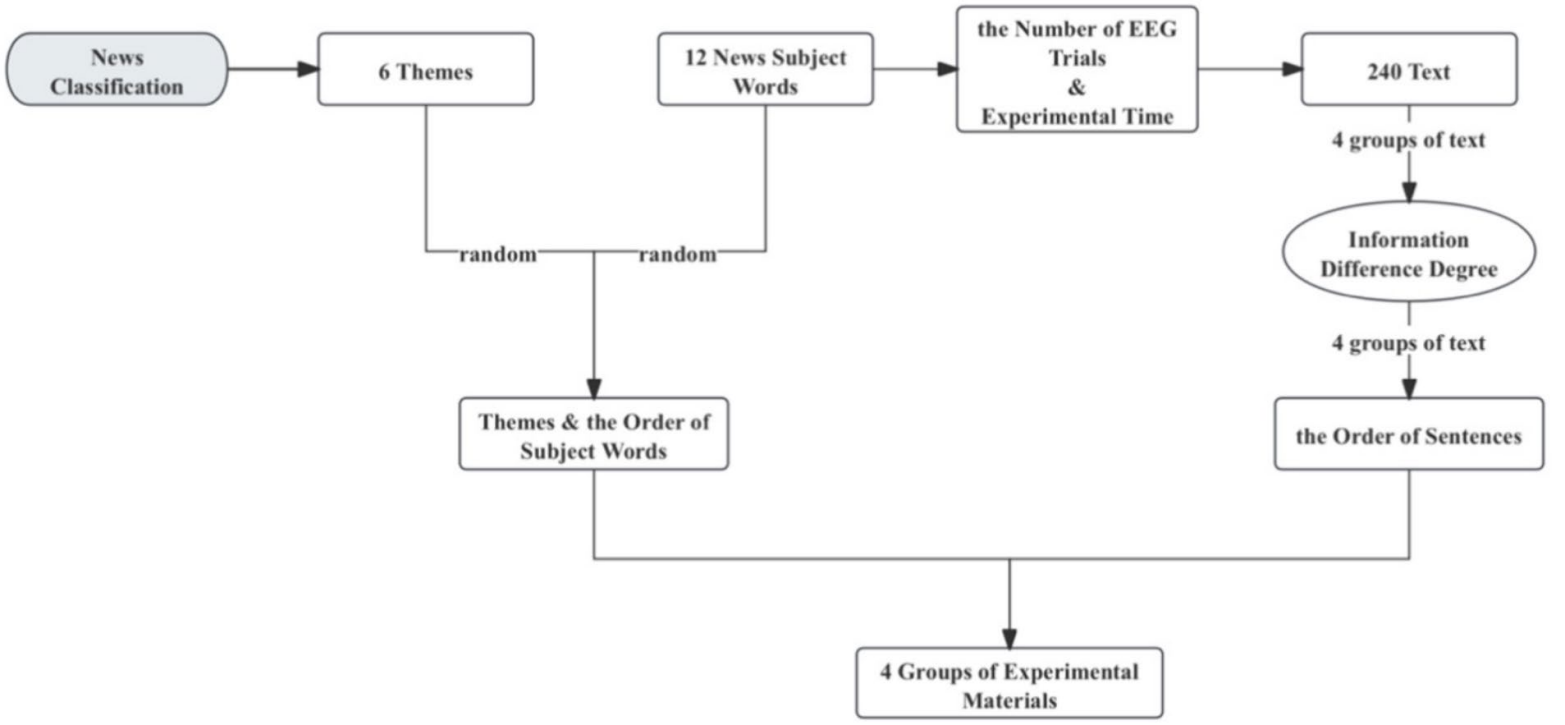
Experimental material acquisition process.

The text under each subject word is randomly arranged in order labeled, and arranged according to Appendix C to reduce the negative influence of not presenting a similar position and obtain the final experimental material. The process of obtaining experimental materials is shown in Figure 2.

As for the selection of long articles, considering the content length and theme consistency of the articles, four articles with appropriate length were selected from Liang Shiqiu’s food prose collection, and then each article was artificially divided into 60 texts to obtain the final experimental material of long articles. See Appendix D for the long article.

## 6 Experimental design

This experiment adopted a single-factor experimental design, with one independent variable (text dissimilarity, 3 levels) and 3 dependent variables (working memory capacity, working memory load, attention resources). The experimental tasks were divided into reading tasks and digital span tasks. Considering the impact of individual differences on EEG signals, each participant needed to complete three reading tasks under the degree of text dissimilarity.

### 6.1 Participant

In this experiment, a total of 27 undergraduates or master’s students were recruited through voluntary registration, ranging from 20 to 25 years old, with an average age of 23.30 (SD = 1.442), including 15 males and 12 females. All participants were in good health, right-handed, and had no psychiatric or neurological history. All subjects are willing to participate in this experiment, each subject needs to participate in 3 experiments. Four participants’ data were excluded from the subsequent analysis since nearly 80% of their experimental data were caused by improperly conducted. Thus, data from 23 participants were used to analyze the working memory load, working memory capacity, and EEG preprocessing.

### 6.2 Experimental tasks

Each participant underwent two tasks of experiments, which were the digital span task and the reading task. Both kinds of experimental tasks need to be carried out by wearing EEG caps.

#### 6.2.1 Digital span tasks

Participants wore EEG equipment and adjusted their posture. When the “+” symbol appeared in the center of the screen, the digital span task began. The “+” duration was set to 2000 ms. A number appeared in the center of the screen for 1 s, which the two consecutive numbers are different, and a total of appeared, but participants were not told the number of numbers and asked to stay still during the 20-number display and remember as many numbers as possible. After the 20 numbers were displayed, the participants were required to immediately write down the numbers they could remember on a piece of paper in the reverse order of the numbers displayed on the screen.

#### 6.2.2 Reading tasks

**Figure 3:**
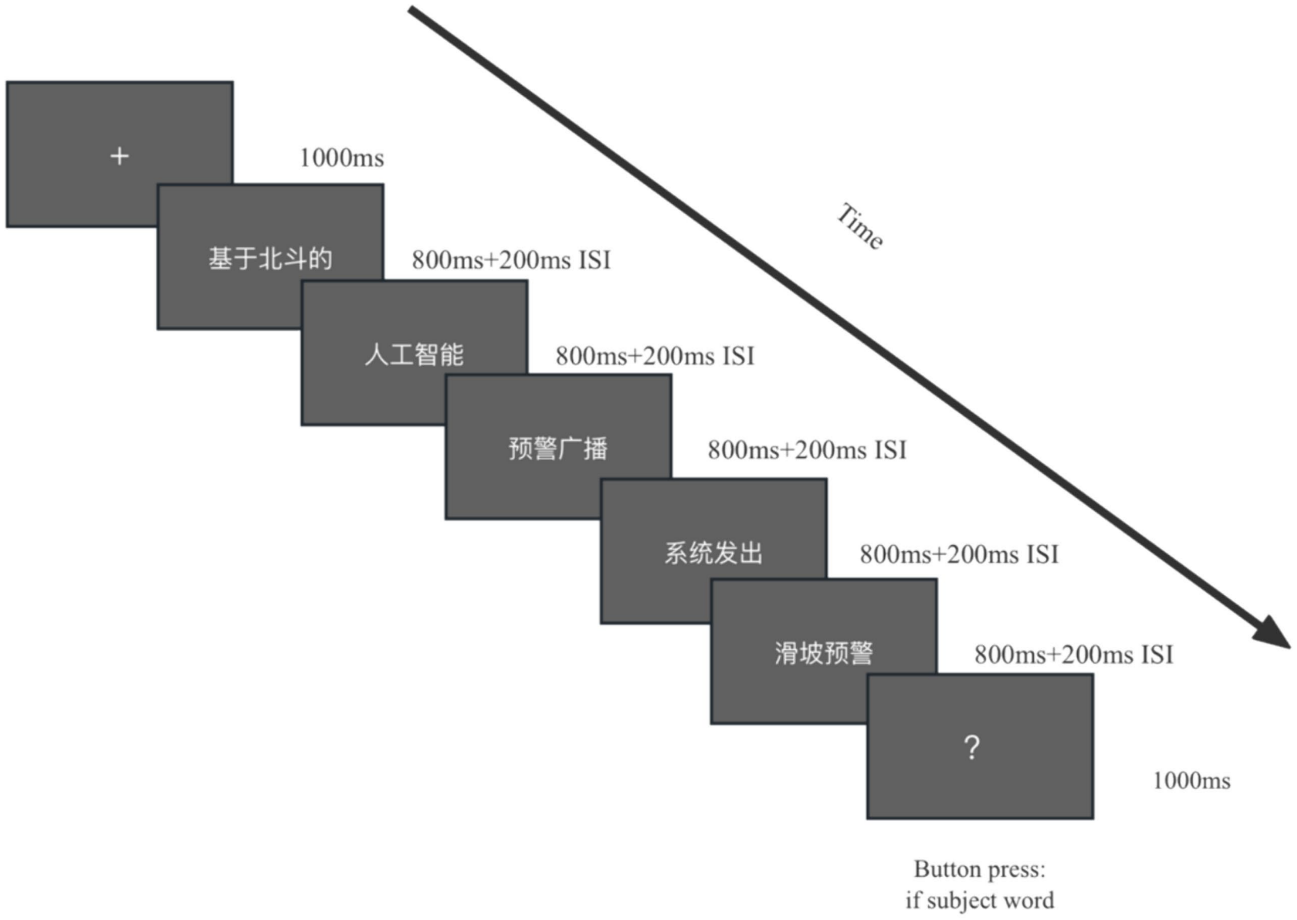
Experimental pattern diagram for one trial of reading tasks.

In the reading task, due to the long length of a text, a text is presented sequentially in the form of multiple words in the center of the screen, to avoid obvious horizontal eye movement artifacts in the EEG signal and induce accurate ERP components. The number of participles in each text is controlled at 5–7, the text in chunks of 3–5 Chinese characters was presented at a time on the screen, as shown in Figure 3.

After the participants adjusted their sitting posture, rest for 2–3 min. The “+” symbol appears in the center of the screen, the duration is 1,000 ms, and a text is presented in sequence. The text content is presented sequentially in the form of multiple words, the word presentation time is 800 ms, and after the completion of a word presentation, a blank screen with a duration of 200 ms appears. After the text content is presented, a “?” appears in the center of the screen. If the subject reads the target theme word that requires the response in this text, press the left mouse button, otherwise, no response will be made. A reading task presents a total of 60 texts, 10 of which contain target subject words (singular stimuli).

### 6.3 Experimental process

The experiment took place in a quiet laboratory, and only one researcher and one participant were present in each experiment. Each participant was asked to complete the entire experiment at three different levels and the experiments were spaced with intervals of at least one day.

**Figure 4:**
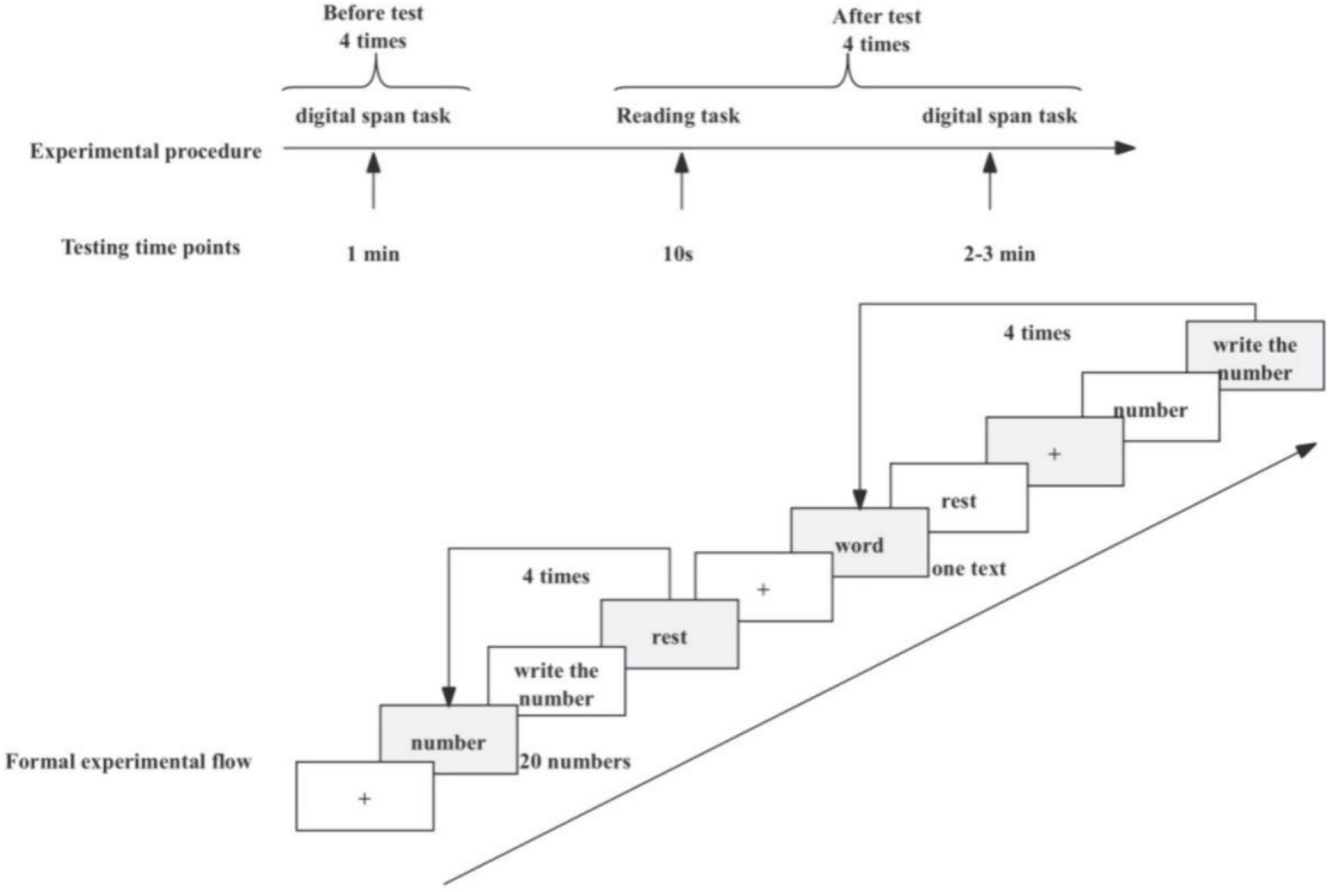
Formal experiment process.

Before the start of the formal experiment, the researchers introduce the background, purpose, process, and precautions of the experiment to each participant. The researchers guided the subjects to sit comfortably on a chair wearing an electrode cap, which needed to adjust the electrode positions to meet the experimental specifications. After confirming the completion of all preparatory work, the formal experiment began. Firstly, the participants needed to complete the task of pre-measuring digital memory span four times, and then they started the reading task after a rest of 2–3 min. Finally, after each reading task, a post-test numerical memory span task should be completed, resulting in a total of four reading tasks to be accomplished, as shown in Figure 4.

### 6.4 EEG recording and preprocessing

In this experiment, the EEG signals were recorded with 18 Ag/AgCl active electrodes (Brain Products, Munich, Germany). The EEG system consists of three main areas: signal recording module, signal amplifier system, and data analysis system. According to the 10–20 International System, the signal recording system contained a ground electrode and a reference electrode, which were placed in the left ear lobe, and this also was mounted within an elastic EasyCap to record EEG data. The amplifier system was used to receive the voltage signal from the electrode and amplify it to a range compatible with the analog-to-digital converter, which then converts the voltage signal from analog to digital. A data analysis system was used to record the signal and convert the format of the input signals. During the experiment, the EEG signal was digitized online with a sampling rate of 1,000 Hz and amplified using a 0.016 Hz–100 Hz A/D bandpass.

The device used to present the stimulus materials and record the EEG data is by Dell desktop computer with a Windows 7 operating system, a 22-inch screen size, and a resolution of 1,366 × 768. One computer ran Eprime3.0 software to display experimental stimuli, and the other computer ran the Brain Vision Recorder software to record and save EEG signals.

For the recorded data, Mne and Jupyter NoteBook were used for preprocessing. The left mastoid was used as the reference electrode. The filter bandpass was 0.1–30 Hz. Power line interference was eliminated by 50 Hz notch filtering, and the artifact components were removed by independent component analysis (ICA) (Ding et al., 2018). The EEG data from the 20 s digital display stage of the digit span task were selected for analysis. The time window was from 0 ms to 2000 ms and divided into baseline for signal correction and reading signal. Filtering was conducted using a finite impulse response (FIR) to select EEG data in the Theta band (4–8 Hz). The fast Fourier transform multitaper method (FFT-MTM) was calculated to the power spectral density values in the theta band. Finally, the sum of these values within the theta band was used as an index to measure the working memory load of participants in the digit span task.

### 6.5 Statistical and analysis

#### 6.5.1 Working memory capacity

The results of the digit span task, which measured working memory capacity, were processed and analyzed using Numpy, Pandas, Matplotlib, and Scipy. In the experiment, a total of four tests were carried out under each condition, and the average score of these tests was used to measure each participant under three conditions (see Table 2). One outlier was identified and removed before proceeding with the subsequent analysis. Because the participants’ states might be different across each experimental session, this research calculated the change in working memory capacity after reading text information by subtracting the working memory capacity before reading. A one-way repeated measures ANOVA was used for differential analysis.

#### 6.5.2 Working memory load

All the EEG data to measure working memory load were processed and analyzed by Numpy, Pandas, Matplotlib, and Scipy. It was found that there were some outliers in the dataset. This is because muscle activity, eye blinks, and electrical noise could affect EEG signals, making it difficult to record and isolate valid data. These invalid EEG data points manifest as outliers and are likely less related to working memory load. Therefore, the outliers were treated as missing data, and if any of the four segments of EEG data under a condition contained missing values, the mean of the remaining valid data will be used as the representative value for the working memory load under that condition.

**Table 2:** Data description of working memory capacity.

The order of the experiment	Levels of text dissimilarity	Count	M	SD	Shapiro	*p*-value
Before	max	23	5.25	1.65	0.960	0.460
	min	23	4.95	2.05	0.976	0.835
	baseline	23	5.14	1.77	0.974	0.772
After	max	23	4.80	1.69	0.971	0.713
	min	23	5.60	1.75	0.977	0.841
	baseline	23	5.91	1.74	0.956	0.385

This study primarily explored changes in working memory load when participants performed the same memory task before and after reading textual content. Therefore, the change in working memory load at each level of text difference is calculated by subtracting the baseline of EEG data from the EEG data recorded during reading at each level of text dissimilarity. A normality analysis was conducted following data processing, and the results could be found in Table 3.

**Table 3:** Data description of the changes on memory load data.

Levels of text dissimilarity	Count	M	SD	Shapiro	*p*-value
max	19	1.97 × 10^−8^	1.97 × 10^−8^	0.959	0.558
min	18	−4.21 × 10^−8^	−4.21 × 10^−8^	0.935	0.242
baseline	20	1.67 × 10^−8^	1.67 × 10^−8^	0.966	0.667

#### 6.5.3 Attentional resources

**Figure 5:**
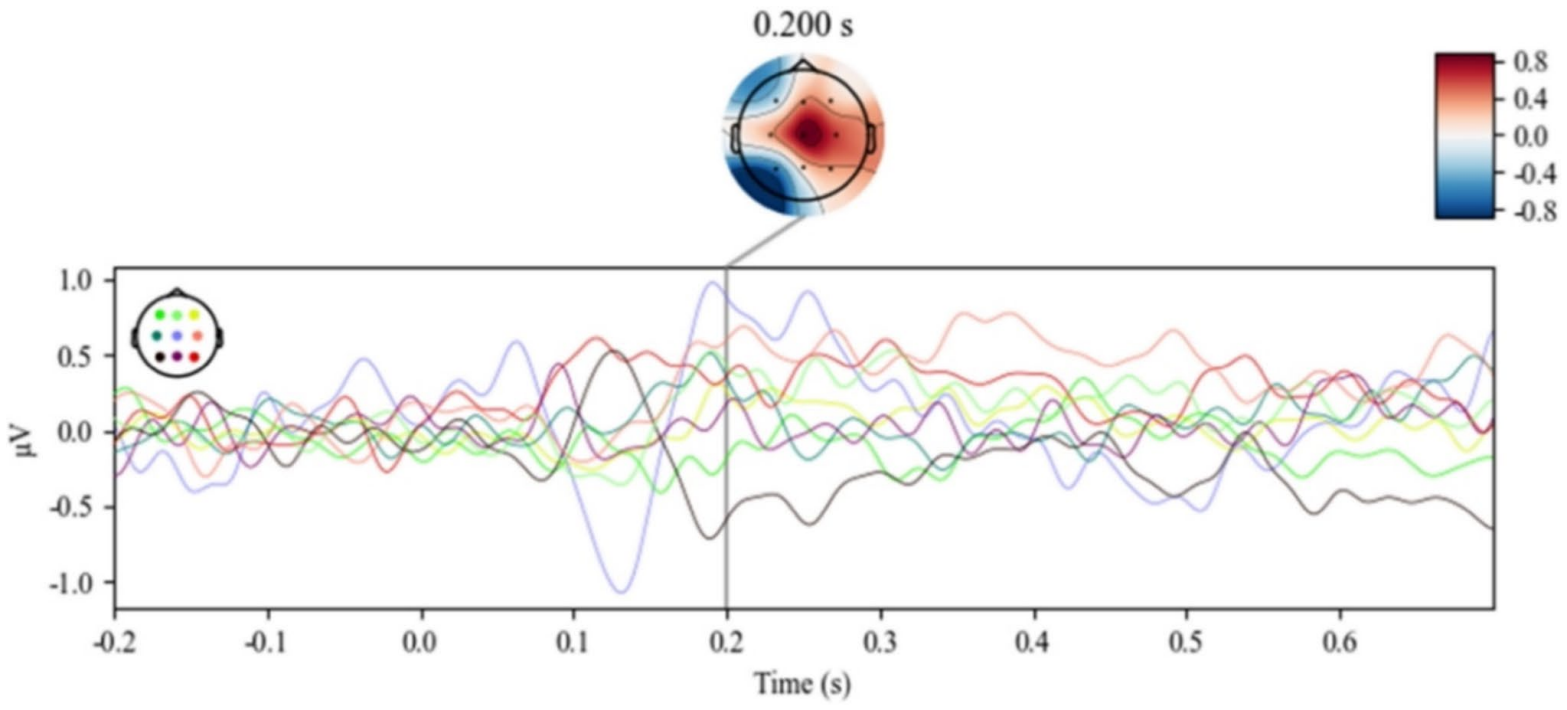
Potential fluctuation diagram of each electrode under the max condition.

ERP data in the reading process was utilized for processing and analysis by Mne, Numpy, Pandas, Matplotlib, and Scipy. ERP data was filtered from each participant at each level of dissimilarity, and then selected segments from 0.2 s before the appearance of the target word to 0.7 s after its appearance for analysis. Finally, removal of artifacts from ERP data recording, the data were averaged to obtain the evoked potentials. The brainwave-evoked data at the maximum distinctiveness are shown in Figure 5.

Although the experimental design was based on the Odd Ball paradigm during reading tasks, the anticipated P300 component was not elicited. This might be attributed that participants might have anticipated the appearance of topic-related keywords, because a sentence was presented in the form of a sequence of words, and the preceding words could potentially serve as cues for the imminent occurrence of the topic-related keywords in the reading task, resulting in shortened reaction times. Therefore, this experiment might lead to a shortened latency of the P300 signal and manifest as the P200 signal.

**Figure 6:**
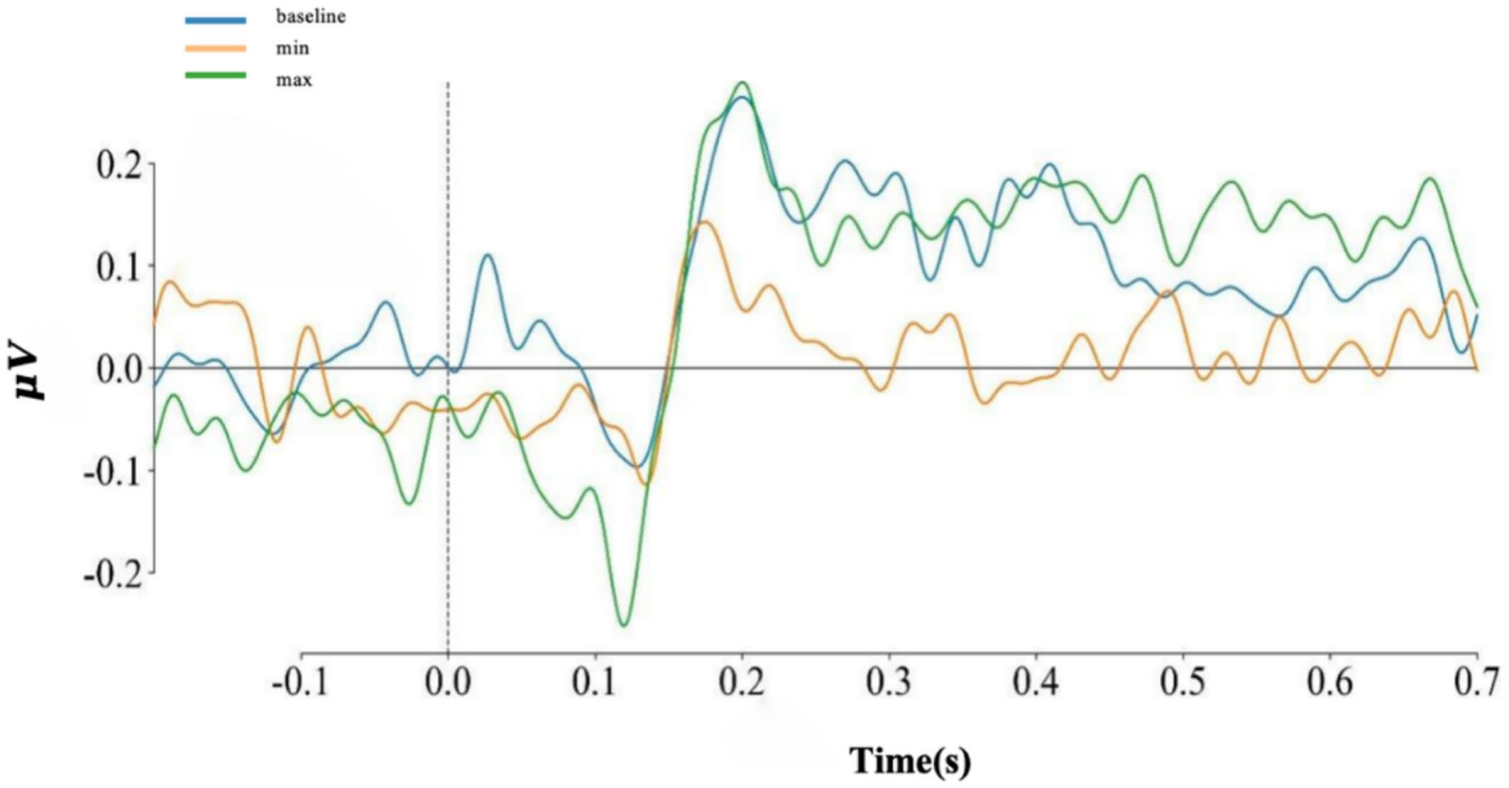
The average of all electrodes at each level.

**Figure 7:**
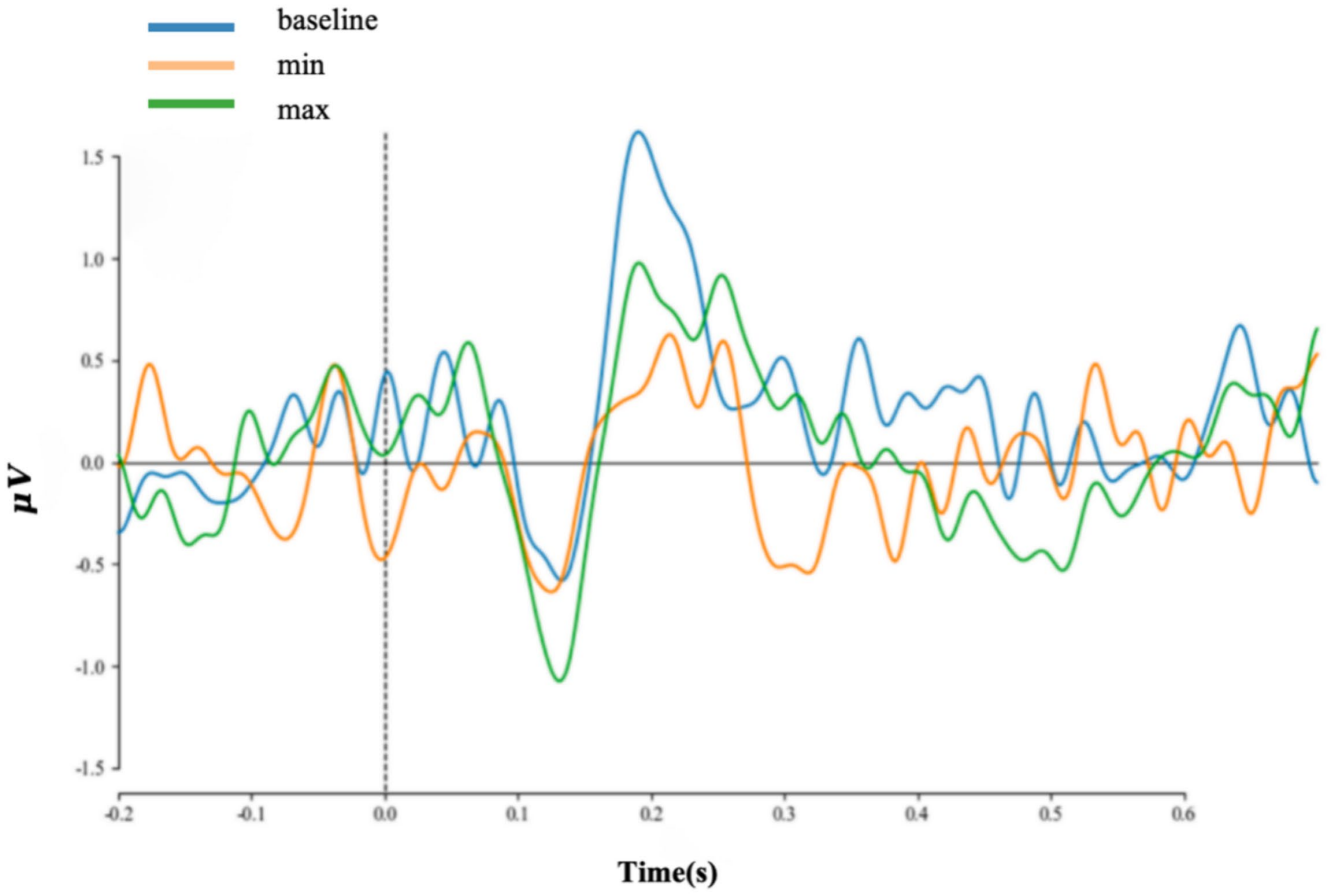
ERP data on Cz electrodes at each level.

For each level of dissimilarity in trials, all electrodes were averaged, as shown in Figure 6, and EEG data at electrode position Cz, as shown in Figure 7. The emergence of the P200 ERP was observed in the reading task. To further investigate the evoked electroencephalographic P200 component in this study, this research analyzed it from two perspectives: latency and amplitude.

**Table 13:** Comparison of experimental results and experimental assumptions.

Hypothesis	Variables	Result	Consistency
H1	Working memory capacity^Δ^	max < min (*p* = 0.016*) max < baseline (*p* = 0.003***) min < baseline (*p* = 0.796)	All assumptions are satisfied
H2	Working memory load^Δ^	max < min (*p* < 0.001*) max > baseline (*p* = 0.791) min < baseline (*p* < 0.001***)	Some of assumptions are satisfied
H3	P200 amplitude	max > min (*p* = 0.080) max < baseline (*p* = 0.005***) min < baseline (*p* = 0.142)	Some of assumptions are satisfied

*p < 0.01, **p < 0.05, and ***p < 0.001. ^Δ^The difference between the measurements before and after.

## 7 Result

The results of the experiment were described as follows:

### 7.1 Working memory capacity

Descriptive statistics for the change in working memory capacity are shown in Table 4. The change in working memory capacity at the three levels has undergone a Shapiro–Wilk normality test. Additionally, the data has passed Mauchly’s sphericity test (*W*_2_ = 0.936; *p* = 0.515). Results from a one-way repeated-measures analysis of variance indicated significant differences (*F*_(2,42)_ = 5.321, *p* = 0.009).

**Table 4:** Data description of changes on working memory capacity.

Levels of text dissimilarity	Count	M	SD	Shapiro	*p*-value
max	22	−0.45	1.29	0.954	0.384
min	22	0.68	1.61	0.947	0.279
baseline	22	0.80	1.14	0.959	0.469

Following the reading texts in the “max” condition, participants exhibited a decrease in working memory capacity (Mean = −0.45; SD = 1.29). However, after reading texts with “min” and “baseline” conditions, there was an increase in working memory capacity (Mean = 0.68; SD = 1.61; Mean = 0.8; SD = 1.14). To further analyze the data, post-hoc analyses were conducted on the change in working memory capacity at the three dissimilarity levels using the least significant difference (LSD). The results are presented in Table 5.

**Table 5:** *Post hoc* analysis of changes in working memory capacity.

Pairwise comparisons	*t*_(21)_	Adj.*p*
max-min	−2.632	0.016^**^
max-baseline	−3.377	0.003***
min-baseline	−0.262	0.796

*p < 0.01, **p < 0.05, and ***p < 0.001. Adj.*p* indicates the *p* value adjusted after analysis.

There was a significant difference in the change in working memory capacity between the different levels “max” and “min,” as well as between “max” and “baseline” (*p* < 0.05). In terms of the change in working memory capacity, the “min” level was higher than the “max” level, and the “baseline” level was higher than the “max” level. However, there was no significant difference in the change in working memory capacity between different “min” and “baseline” levels (*p* = 0.796, min_mean_ = 0.68, baseline_mean_ = 0.80).

### 7.2 Working memory load

Descriptive statistics for the change of working memory load, which is calculated by the PSD of theta band, are shown in Table 6. A one-way repeated measures ANOVA was used for different analyses. The change in working memory load at the three levels has undergone a Shapiro–Wilk normality test, and the sample of each condition obeyed the normal distribution. Additionally, the data has passed Mauchly’s sphericity test (*W*_2_ = 0.797; *p* = 0.093). Results from a one-way repeated-measures analysis of variance indicated significant differences (*F*_(2,44)_ = 16.044, *p* < 0.001). The result exhibited an increase in working memory load after reading texts with levels of “max” and “baseline.” However, after reading texts with a level of “min,” their working memory load decreased. Further post-hoc analysis was conducted and shown in Table 7.

**Table 6:** Data on changes in working memory load (filling missing values).

Levels of text dissimilarity	Count	M	SD	Shapiro	*p*-value
max	23	1.97 × 10^−8^	3.68 × 10^−8^	0.952	0.319
min	23	−4.21 × 10^−8^	5.69 × 10^−8^	0.965	0.568
baseline	23	1.67 × 10^−8^	4.50 × 10^−8^	0.974	0.782

**Table 7:** *Post hoc* analysis of changes in working memory load.

Pairwise comparisons	*t*_(22)_	Adj.*p*
max-min	4.190	<0.001***
max-baseline	0.268	0.791
min-baseline	−5.741	<0.001***

*p < 0.01, **p < 0.05, and ***p < 0.001. Adj.*p* indicates the *p* value adjusted after analysis.

The results indicated significant differences in the changes of working memory load values between different levels of “max” and “min,” as well as between “min” and “baseline” (*p* < 0.05). In terms of the change in working memory load, the “max” level was 147% higher than the “min” level, and the “baseline” level was 140% higher than the “min” level. However, there is no significant difference in the change in working memory load between different levels of “max” and “baseline” conditions (*p* = 0.791).

### 7.3 Attentional resources

#### 7.3.1 Amplitude

Descriptive statistics for the average amplitude of the P200 component under three experimental conditions are shown in Table 8. A one-way repeated measures ANOVA is used to test for significant differences, and post-hoc analyses are shown in Table 9.

**Table 8:** Descriptive statistics for mean amplitude of P200 (filling missing values).

Levels of text dissimilarity	Count	M	SD	Shapiro	*p*-value
max	23	1.07	0.91	0.972	0.742
min	23	0.49	1.05	0.967	0.626
baseline	23	1.6	1.46	0.974	0.791

**Table 9:** Pairwise comparisons of *post hoc* analyses of mean amplitudes of P200.

Pairwise Comparisons	*t*_(22)_	Adj.*p*
max-min	1.834	0.080*
max-baseline	−1.522	0.142
min-baseline	−3.095	0.005***

*p < 0.01, **p < 0.05, and ***p < 0.001. Adj.*p* indicates the *p* value adjusted after analysis.

The average amplitude of the P200 component at the three levels has undergone a Shapiro–Wilk normality test. Additionally, the data has passed Mauchly’s sphericity test (*W*_2_ = 0.982; *p* = 0.828). Results from a one-way repeated-measures analysis of variance indicated significant differences (*F*_(2,44)_ = 5.295, *p* = 0.009).

Descriptive statistics for the peak amplitude of the P200 component under three experimental conditions are given in Table 10, and post-hoc analyses are shown in Table 11.

**Table 10:** Descriptive statistics of peak amplitudes of P200 components (filling missing values).

Levels of text dissimilarity	Count	M	SD	Shapiro	*p*-value
max	23	1.43	1.1	0.98	0.912
min	23	1.09	1.12	0.946	0.239
baseline	23	2.46	1.32	0.97	0.685

**Table 11:** Pairwise comparisons of post-hoc analyses of peak amplitudes of P200 components.

Pairwise comparisons	*t*_(22)_	Adj.*p*
max-min	0.953	0.351
max-baseline	−3.094	0.005**
min-baseline	−3.714	0.001***

*p < 0.01, **p < 0.05, and ***p < 0.001. Adj.*p* indicates the *p* value adjusted after analysis.

The peak amplitude of the P200 component at the three levels has undergone a Shapiro–Wilk normality test. Additionally, the data has passed Mauchly’s sphericity test (*W*_2_ = 0.987; *p* = 0.873). Results from a one-way repeated-measures analysis of variance indicated significant differences (*F*_(2,44)_ = 8.226, *p* = 0.001).

#### 7.3.2 Latency

Descriptive statistics for latency of the P200 component under three experimental conditions are given in Table 12.

**Table 12:** Descriptive statistical table of the latent period of P200 components.

Levels of text dissimilarity	Count	M	SD	Shapiro	*p*-value
max	23	199.22	12.86	0.927	0.093
min	23	199.7	13.68	0.913	0.048
baseline	23	196.74	12.28	0.936	0.147

The latency data of the P200 component at the min level did not pass the Shapiro normality test, so non-parametric testing methods should be employed. Results from the Friedman two-way rank analysis of variance indicated no significant differences in the latency of the P200 component among the three levels (*M*_(3,23)_ = 1.82, *p* = 0.403).

## 8 Discussion

The present study aimed to explore the impact of fragmented reading behavior on cognitive ability through the use of ERP components. There were three main findings in this study (see Table 13).

The first finding is that, compared with reading the text content with a lower degree of text dissimilarity, reading higher leads to less working memory capacity when performing subsequent cognitive tasks. The result showed that after reading the content with a higher degree of text dissimilarity, the participant had a lower digital span task performance. This might be attributed to a higher frequency of cognitive task switching when reading content with a higher degree of text dissimilarity, which consumes more cognitive resources and affects the executive control function. Meanwhile, the executive control function is positively correlated with working memory capacity and reflected in low performance on digital-span tasks.

In particular, concerning the degree of change in working memory capacity, the results of our study suggest that working memory capacity has the highest degree of decline in the ‘max’ condition, where excessive attention switching process might exert a significant negative impact on executive control function. Moreover, there was little difference in the degree of change in working memory capacity between the ‘min’ condition and the ‘baseline’ condition, and there was also no significant decline.

Secondly, the experimental results suggest that the working memory load of the min level in subsequent memory tasks is lower than that of the max level and baseline level. However, an apparent contradiction emerges as the working memory load in the subsequent memory task under the lower level in the baseline condition is higher than that in both the “max” and “min” levels. This might be explained by the continuous maintenance of a unified mental representation. When reading long articles, the interconnectedness leads to fewer cognitive switches and more stable cognitive resources. And when transitioning to the digit span task, reallocating cognitive resources might become more challenging, and increase by working memory load. In contrast, after reading fragmented text content, an elevation in working memory load is observed at the “max” level. Fragmented reading lacks the continuous association between preceding and subsequent text, making it difficult to maintain a consistent mental representation over an extended period. The frequent occurrence of cognitive task switches during fragmented reading might reflect a negative impact on executive control functions, requiring more effort during the execution of the digit span task.

The last finding is that the average amplitude of P200 is higher than the ‘max’ and ‘min’ levels in the process of reading long articles, which might be attributed to the interconnectivity between text contents and little switching between cognitive, thereby resulting in reduced consumption of cognitive resources. Notably, when reading fragmented text content, the P200 average amplitude at the “min” level is lower than that at the “max” level, and reading text content at the lower level consumes more cognitive resources. Considering the experimental results on working memory capacity, the lower P200 average amplitude at the “min” level might reflect reduced neural activity that needs to be inhibited during the appearance of odd stimuli (theme words) in a paradigm reminiscent of the Odd Ball experiment. The diminished neural activity implies smaller deviations in the EEG potential amplitude, suggesting the potential reuse of previously activated neurons associated with the encoding and processing of the current text.

However, the results also indicate that after the lower text dissimilarity in reading text content, the participants in subsequent cognitive tasks had more working memory capacity, lower working memory load, and less negative impact on cognitive ability; simultaneously, it also means that even when reading the same text content, the difference degree of text information could be reduced by altering its presentation order, and this could mitigate the negative impact of fragmented reading behavior on cognitive ability, including executive control function and working memory.

Three limitations of this research should be noted. First, although we have taken measures to make the fixed number of experimental tasks, the individual characteristics in work memory capacity might have an impact on our results. In this study, the short sentences and selective themes were identified based on the requirement of the ERP experiment. However, an article might be longer and more complete. At the same time, during the period of preparation for the experiment, some participants might already have read some same materials in the experiment through online news, which would cause them to no longer have a desire to read. Then, each individual required the same attentional resources and working memory in cognitive processing. However, each participant might engage in different mechanisms for the same external information processing due to their knowledge backgrounds, and familiar content might have a better understanding of cognitive processing. Finally, experimental materials in cognitive research consisted of relatively short texts, which might not match the online reading scenarios.

## 9 Conclusion

Based on the attention mechanism of humans, this study examined the cognitive responses associated with fragmented reading behavior. There were two main findings derived from the experiment. Firstly, attention switching, caused by differences in conceptual cognition or working memory representation of text content, is likely the key factor affecting cognitive ability. Secondly, attention switching in fragmented reading behavior might harm cognitive abilities, including executive control and working memory, and reading text with a lower degree of dissimilarity is associated with a smaller negative impact on the cognitive abilities required for subsequent cognitive tasks.

In summary, this paper proposes a method to measure attention switching based on the brain’s attention mechanism through quantitative analysis. The cognitive experiment is utilized to investigate how fragmented reading behavior on new media platforms affects individuals’ cognitive abilities. The research findings could be applied to the design of new media interfaces to provide interventions and offer a reference for reducing the negative impact of fragmented reading behavior on cognitive abilities. Meanwhile, attention switching, resulting from disparities in conceptual cognition or memory representation between preceding and subsequent textual content, does occur when reading fragmented text, and it also could be used by changing the sequence of the reading content to mitigate the negative influence of working memory and executive control function. Additionally, brain activity patterns might differ between long articles and fragmented reading, and the explanation could not only rely on the level of text dissimilarity as a single factor variable. Therefore, future research might need to delve deeper into fragmented reading behavior by observing brain activity patterns.

## Data availability statement

The data presented in the study has been deposited in the following repository: https://pan.baidu.com/s/1il4mV8f8aLy2L6x2Ym1KPg, accession number 17m9.

## Ethics statement

The studies involving humans were approved by Ethics Committee of Chongqing University. The studies were conducted in accordance with the local legislation and institutional requirements. The participants provided their written informed consent to participate in this study.

## Author contributions

JC: Writing – original draft, Writing – review & editing. JL: Writing – original draft, Writing – review & editing. JZ: Writing – original draft, Writing – review & editing. YJ: Writing – review & editing.
