# Supplementary material for: Attention switching through text dissimilarity: a cognition research on fragmented reading behavior
Source: Front Hum Neurosci. 2024 Jun 25;18:1402746. doi: 10.3389/fnhum.2024.1402746 (PMC11231079; doi:10.3389/fnhum.2024.1402746)
Supplement: Supplementary file 1 [file Presentation_1.pdf]

Supplementary Material

1.1 Supplementary Figures

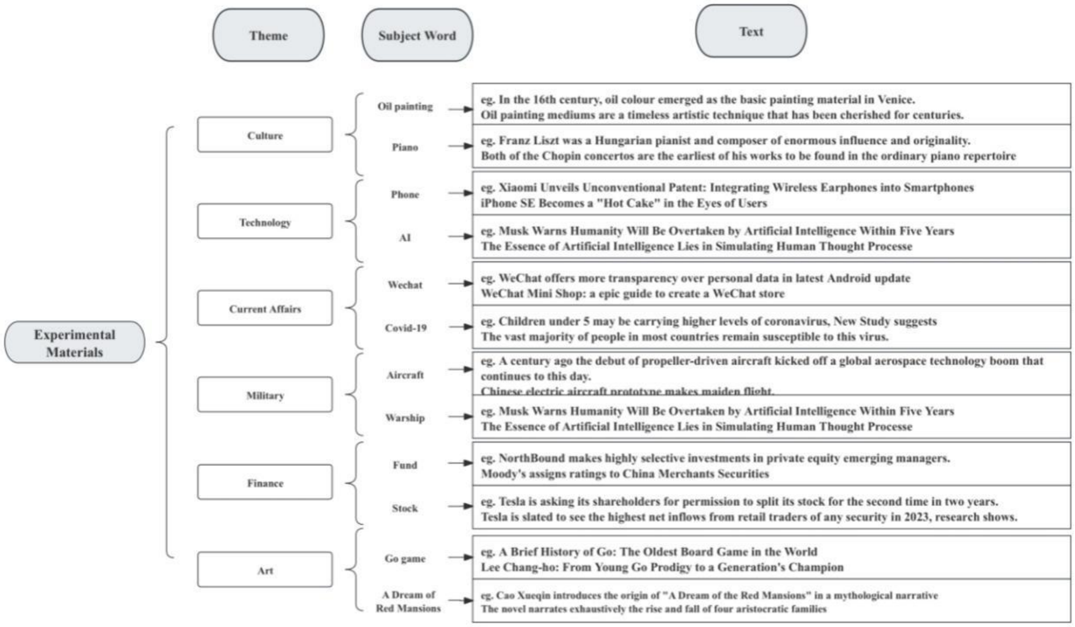

Supplementary Figure 1. Some examples of the English version of texts

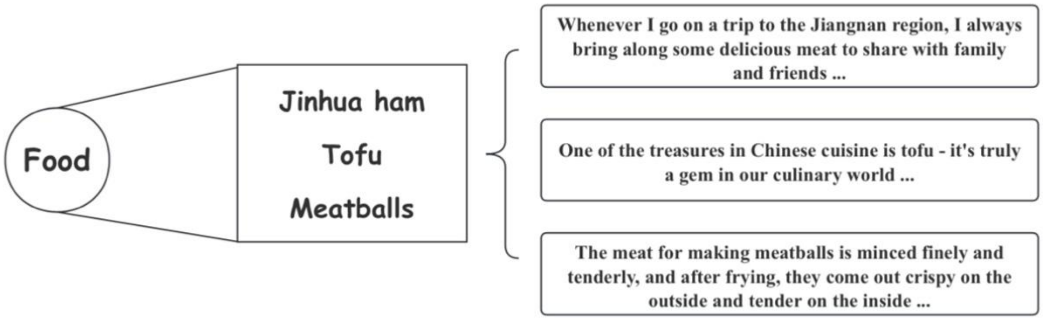

Supplementary Figure 2. Some examples of the texts of long articles with English version
