# Supplementary material for: Attention switching through text dissimilarity: a cognition research on fragmented reading behavior
Source: Front Hum Neurosci. 2024 Jun 25;18:1402746. doi: 10.3389/fnhum.2024.1402746 (PMC11231079; doi:10.3389/fnhum.2024.1402746)
Supplement: Supplementary file 2 [file Table_1.pdf]

Table A1 the English version of themes and subject words

theme	the mark of theme	The first and the third of subject words	The second and the forth of subject words
Technology	A	Phone	Artificial Intelligent
Culture	B	Go game	A Dream of Red Mansions
Current Affairs	C	WeChat	Covid-19
Military	D	Aircraft	Warship
Finance	E	Fund	Stock
Art	F	Oil painting	Piano
