# Supplementary material for: Attention switching through text dissimilarity: a cognition research on fragmented reading behavior
Source: Front Hum Neurosci. 2024 Jun 25;18:1402746. doi: 10.3389/fnhum.2024.1402746 (PMC11231079; doi:10.3389/fnhum.2024.1402746)
Supplement: Supplementary file 3 [file Table_2.pdf]

Table B1 the English version of themes and subject words

order	condition	the mark of text									
1-10	max	A1	B1	C1	D1	**E1**	F1	B2	A2	C2	D2
	min	A1	B1	A2	A3	**E1**	F1	B2	A4	A5	F2
11-20	max	F2	**E2**	A3	B3	D3	F3	A4	C3	B4	**E3**
	min	F3	**E2**	A6	B3	F4	A7	F5	A8	B4	**E3**
21-30	max	F4	D4	C4	A5	**E4**	F5	D5	C5	B5	**E5**
	min	F6	A9	F7	F8	**E4**	A10	F9	F10	B5	**E5**
31-40	max	F6	A6	D6	C6	F7	A7	**E6**	B6	D7	C7
	min	D1	C1	D2	D3	C2	C3	**E6**	B6	D4	C4
41-50	max	F8	**E7**	A8	B7	C8	**E8**	A9	F9	D8	C9
	min	C5	**E7**	D5	B7	C6	**E8**	D6	C7	D7	C8
51-60	max	B8	A10	**E9**	F10	D9	B9	C10	B10	D10	**E10**
	min	B8	C9	**E9**	C10	D8	B9	D9	B10	D10	**E10**
